# Depletion of the diabetic gut microbiota resistance enhances stem cells therapy in type 1 diabetes mellitus

**DOI:** 10.7150/thno.44113

**Published:** 2020-05-17

**Authors:** Wanqi Lv, Dana T. Graves, Linhai He, Yan Shi, Xuliang Deng, Yajun Zhao, Xian Dong, Yi Ren, Xinhua Liu, E Xiao, Yi Zhang

**Affiliations:** 1 Department of Oral and Maxillofacial Surgery, Peking University School and Hospital of Stomatology, 22 Zhongguancun Nandajie, Haidian District, Beijing 100081, People's Republic of China; 2 Department of Periodontics, School of Dental Medicine, University of Pennsylvania, Philadelphia, PA, USA; 3 Peking University Hospital of Stomatology First Clinical Division, 37 Xishikudajie, Xicheng District, Beijing 100034, People's Republic of China; 4 Institute for Immunology and Department of Basic Medical Sciences, Beijing Key Lab for Immunological Research on Chronic Diseases, School of Medicine; Tsinghua-Peking Center for Life Sciences, Tsinghua University, Beijing 100084, People's Republic of China; 5 Department of Geriatric Dentistry, National Engineering Laboratory for Digital and Material Technology of Stomatology, Beijing Laboratory of Biomedical Materials, Peking University School and Hospital of Stomatology, Peking University, Beijing 100081, People's Republic of China; 6 Department of Pediatric Dentistry, Peking University School and Hospital of Stomatology, 22 Zhongguancun Nandajie, Haidian District, Beijing 100081, People's Republic of China; 7 The First People's Hospital of Jinzhong, ShanXi Province 030600, People's Republic of China

**Keywords:** Type 1 diabetes mellitus, Stem cell transplantation, Broad-spectrum antibiotics, Gut microbiota translocation, *Bifidobacterium*

## Abstract

Microbiome, considered as the “second genome” of the host, is altered in type 1 diabetes mellitus (T1DM) patients to a state of dysbiosis. Mesenchymal stem cell (MSC) transplantation is a promising treatment for T1DM but is limited by several factors in the diabetic host. In this study, we tested the hypothesis that dysbiotic gut microbiota may limit MSC therapy, and modulating gut microbiota may help to improve the effects of MSC transplantation.

**Methods:** NOD/Ltj mice, treated with adipose-derived stem cells (ADSCs), were fed with an antibiotics cocktails (Abx) for 1 week. The blood glucose levels, insulitis, intestinal permeability and gut bacteria translocation to the pancreas were evaluated. 16s rRNA and colon tissue transcription sequencing were performed to analyze beneficial bacteria and reactive host biomolecules in the ADSCs+Abx group. Based on the sequencing results, specific bacteria were gavaged orally to diabetic mice to confirm their effect on ADSCs transplantation in T1DM was determined.

**Results:** We found that the recolonized the diabetic gut microbiota abolished the therapeutic effect of ADSCs. On the contrary, depletion of the diabetic gut microbiota by antibiotics treatment in diabetic mice significantly enhanced the therapeutic effects of ADSCs as measured by reversal of hyperglycemia, insulitis, and increased insulin output. Mechanistically, treatment with antibiotics increased the abundance of *Bifidobacterium* in the gut and reduced bacterial translocation to the pancreas by promoting Mucin2 expression and thickening the mucus layer through TRPM7. The mechanism was confirmed the re-colonization of the gut by *B.breve* through oral gavage that produced similar results.

**Conclusions:** These results provide the rationale for a new approach to improve MSC therapy for T1DM by altering the gut microbiota.

## Introduction

The mammalian gastrointestinal tract harbors diverse and active microbial communities. The dynamic cross-talk between the host and its microbiota serves as another “host system” that maintains homeostasis to resist pathologic changes or block therapeutic treatments 1, 2. In some cases, perturbation of the healthy microbiota results in dysbiosis and accelerates disease development, while in others, correcting the dysbiotic microbiota may be therapeutic 3-6. Type 1 diabetes mellitus (T1DM), also known as autoimmune diabetes, is characterized by insulin deficiency, which results from the destruction of β cells in the pancreas 7, 8. Accumulating evidence links gut microbiota to T1DM. Infants, who later are stricken with T1DM have a pre-diabetic intestinal microbiota that is characterized by fewer* Bifidobacterium spp.* and elevated levels of *Bacteroides spp*. 9. After the onset, patients with T1DM exhibit a less diverse and less stable gut microbiome compared with healthy controls 10, 11. Similarly, the development of T1DM in non-obese diabetic mice is also affected by microbial changes in the gut 3, 12. All these data suggested a clear connection between gut microbiota and onset or acceleration of T1DM.

The mechanisms that underlie the intestine-pancreas axis have recently been reported. Immune dysregulation secondary to gut microbial dysbiosis has been proposed as a contributor to autoimmunity in T1DM 13, 14. Besides, the dysbiosis of gut microbiota can also affect the intestinal barrier function. It has been proposed that an impaired intestinal barrier function in T1DM may increase the presence of the gut microbiota in the systemic circulation and, in turn, cause dysfunction of islet cells 15, 16. Many factors may contribute to the impaired barrier function with one possibility being a breakage of the mucus layer integrity in pre-diabetic NOD mice compared with control strains 16. It is also possible that bacteria directly translocate to the draining lymph nodes of the pancreas to accelerate the development of autoimmune T1DM 17. Both reports suggest that ectopic bacterial translocation caused by an impaired gut barrier may be conducive to T1DM.

Mesenchymal stem cell (MSC)-based therapy has shown promising prospects in the treatment of many autoimmune diseases, including diabetes 18. In streptozotocin (STZ) induced T1DM mice model, MSC transplantation alleviated the hyperglycemia by reducing insulitis and increasing plasma and islets insulin contents 19, 20. However, despite the anecdotal success there is no approved stem cell therapy for T1DM due to the unsatisfactory clinical trials 21, 22 with the recognition that host factors play a critical role in the disappointing success rates. Because of the close relationship between T1DM and intestinal microbiota, we hypothesized that the diabetic gut microbiota affects outcomes, although causal relationships and potential mechanisms have not been reported 23.

In this study, we explored the role of host dysbiotic intestinal microbiota in stem cell therapy by the transplantation of adipose-derived stem cells (ADSCs).

## Results

### Therapeutic effects of ADSCs transplantation are abolished by diabetic gut microbiota re-colonization

**Figure 1:**
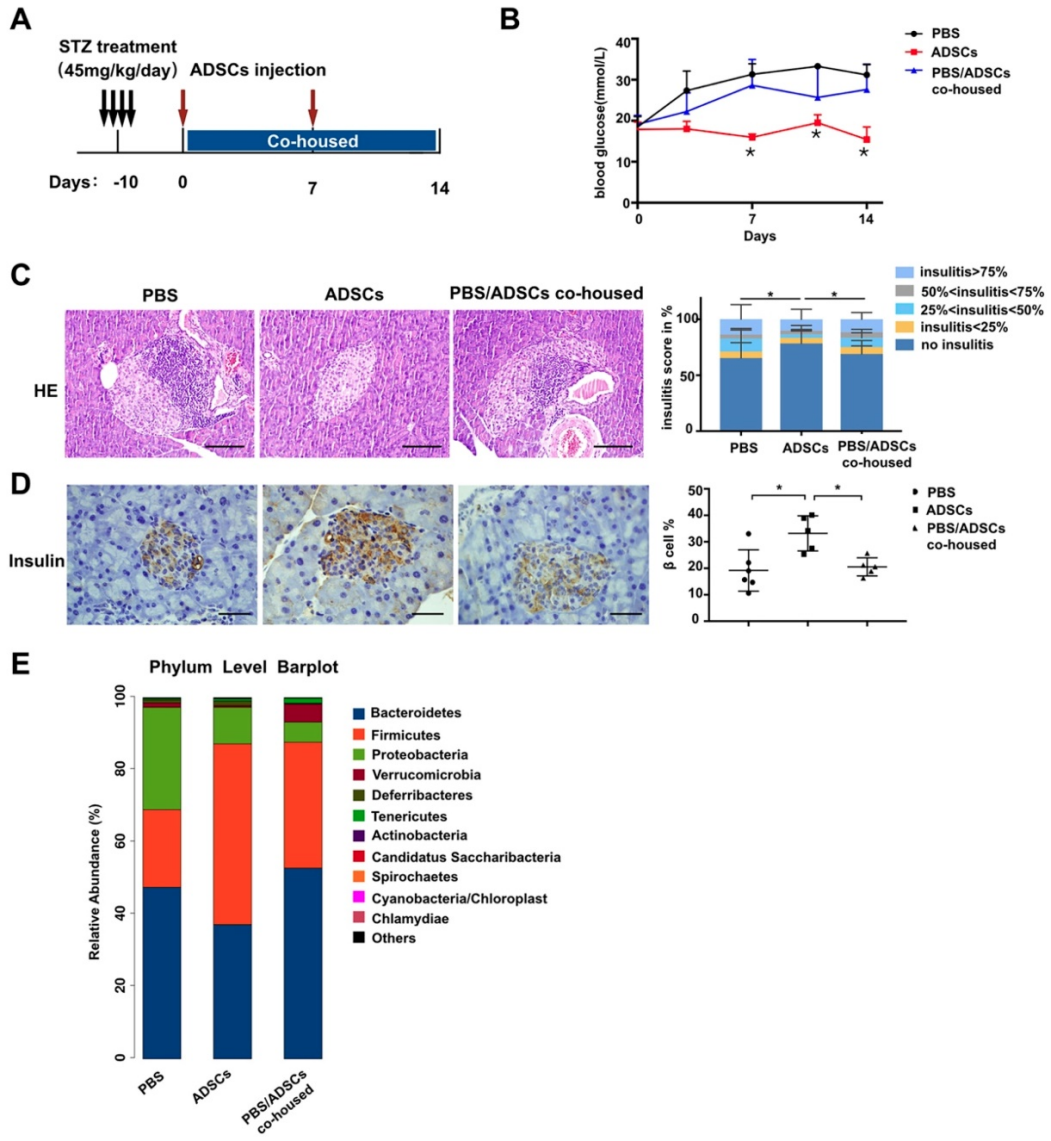
** Gut microbiota participates in the therapeutic effect of ADSCs transplantation in T1DM mice.** (A) The treatment schedule for STZ, ADSCs transplantation. (B) Blood glucose levels (mmol/L) were measured twice a week after ADSCs transplantation for 14 days (N=11-14). (C) H&E staining of pancreas at 2 weeks post ADSCs transplantation; insulitis score was evaluated at right. N=5-6, Scale bar: 100 μm. (D) Insulin immunohistochemistry staining showing β cells preserved in islets. The positive insulin staining area (%) in pancreatic islets was quantified using the image Pro Plus at right, N=5-6, Scale bar: 40 μm. (E) Gut microbiota composition evaluated by 16s rRNA sequencing, N=7-12. Data is presented as Mean ± SEM. *P<0.05, **P<0.01.

To accelerate T1DM development, NOD mice were treated with STZ according to the timeline in Figure 1A. After mice developed diabetes, one group of mice was transplanted with ADSCs through the tail vein, and the other group received PBS as control. As expected, the levels of blood glucose in ADSCs transplantation group were significantly lower than that in PBS group at 14 days post transplantation (21.46 ± 2.163 mmol/L vs 29.65 ± 1.514 mmol/L, p=0.0074, Figure 1B). Furthermore, histological analysis of pancreatic islet inflammation (degree of insulitis as scored on representative HE stained islets shown in Figure S1A) revealed a higher proportion of islets without insulitis. And more β cell preservation in the ADSCs group also confirmed a better therapeutic effect of ADSCs transplantation (Figure 1C-D).

However, the reduced blood glucose levels in ADSCs-treated mice were reversed after co-housing these mice with PBS treated mice. Co-housed mice acquire each other's gut microbiota because they are coprophagic; therefore through co-housing ADSCs mice could receive the gut microbiota from PBS-treated diabetic mice. These results were also supported by increased insulitis scores and decreased β cell preservation in the PBS/ADSCs co-housed group compared with the ADSCs group (Figure 1C-D).

**Figure 2:**
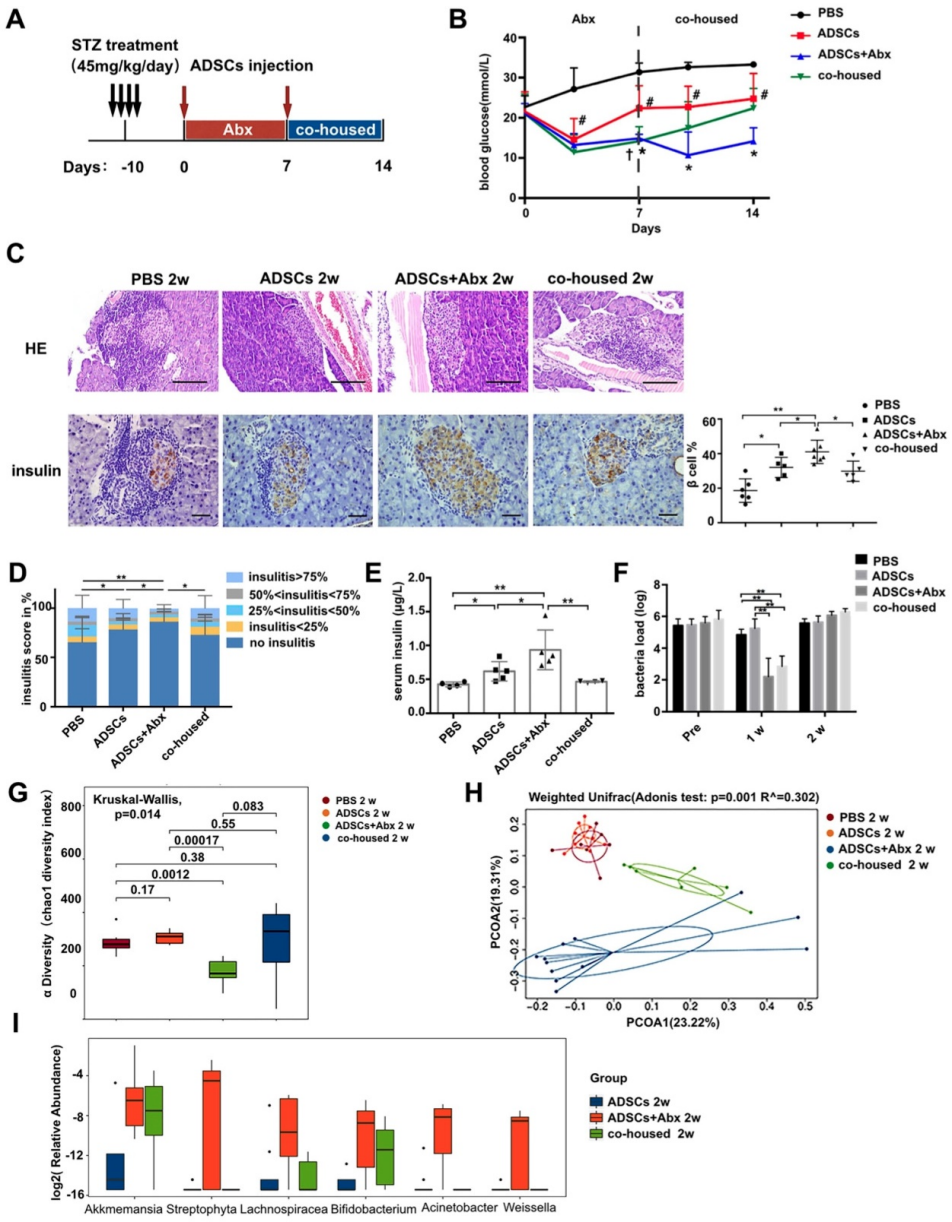
** ADSCs+Abx treatment further reduces insulitis and preserves β cell function by altering the gut microbiota.** (A) The treatment schedule for STZ, ADSCs transplantation and Abx water feeding. (B) Blood glucose levels (mmol/L) were measured twice a week after ADSCs transplantation for 14 days (N=6-8). *P<0.05, ADSCs vs ADSCs+Abx; ^#^P<0.05, PBS vs ADSCs; ^†^P<0.05, ADSCs vs co-housed. (C) H&E staining of pancreas at 2 weeks post ADSCs transplantation, N=5-6, Scale bar: 100 μm; insulin immunohistochemistry staining showing β cells preserved in islets. The positive insulin staining area (%) in pancreatic islets was quantified using the image Pro Plus at right, N=5-6, Scale bar: 40 μm. (D) Insulitis score in pancreatic islets. (E) Serum insulin concentration, N=4-5. (F) Load of mice fecal bacteria detected by real-time PCR assay using a standard curve at 2 weeks post ADSCs transplantation. (G) Alpha diversity of mice fecal microbiota was assessed at 2 weeks post ADSCs transplantation, N=6-9. (H) Beta diversity (Unweighted Unifrac) of mice fecal microbiota was assessed at 2 weeks post ADSCs transplantation, N=7-12. (I) Boxplot of fecal bacteria from ADSCs+Abx, ADSCs and co-housed group. Data is presented as Mean ± SEM. *P<0.05, **P<0.01.

To confirm our hypothesis, we then performed 16s rRNA sequencing, and found that ADSCs-treated mice had a different composition of gut microbiota from the diabetic microbiota in PBS-treated mice (Figure 1E), while co-housing reduced these differences. However, the changes in gut microbiota induced by ADSCs transplantation were limited. As shown in Figure 2G-H, ADSCs transplantation alone did not change the diversity of diabetic gut microbiota. These data indicated that dysbiotic gut microbiota might resist the therapeutic effects of ADSCs transplantation. Threrfore, reducing the resistance of dysbiotic gut microbiota might be an alternative approach to enhance the therapy.

### Antibiotic treatment enhances ADSCs therapy by altering the gut microbiota

To test this hypothesis, we applied broad spectrum antibiotics (Abx) treatment to deplete the diabeitc gut microbiota during ADSCs transplantation (Figure 2A). Mice fed with Abx water experienced a transient weight loss but otherwise showed no significant differences from other groups after Abx withdrawal (Figure S1B). Abx treatment significantly improved the impact of ADSCs on blood glucose levels at day 7 and day 14 compared to the ADSCs treatment alone (14.87 ± 0.380 mmol/L vs 22.39 ± 1.981 mmol/L, p=0.004, post-7 days; 14.13 ± 1.386 mmol/L vs 24.76 ± 2.386 mmol/L, p=0.0036, post-14 days, Figure 2B). Abx-treated mice that received ADSCs therapy had improved glucose levels initially, which were not observed if these mice were co-housed with diabetic mice that only had ADSCs therapy without Abx treatment (Figure 2B). The ADSCs/Abx-treated mice re-acquired the diabetic dysbiotic gut microbiota under cohousing conditions based on their coprophagy character. Control experiments demonstrated that the combination of Abx treatment plus ADSCs therapy was responsible for the reversal of the diabetic condition rather than Abx alone (Figure S1C) or individual components of Abx combined with ADSCs (Figure S1D). This was further supported by the examination of the pancreas, which indicated that the proportion of islets without insulitis was higher in the Abx plus ADSCs group compared to ADSCs alone (Figure 2C-D). Immunohistochemistry analysis revealed that Abx treatment plus ADSCs had significantly higher preservation of insulin staining β cell compared with the other groups and higher serum insulin levels compared with the other groups (Figure 2C and 2E).

The above results strongly suggested that alteration in the dysbiotic gut microbiota greatly promoted the successful ADSCs therapy in T1DM. To demonstrate this more conclusively, we performed 16S rRNA real-time PCR and sequencing to identify key constituents of the bacterial communities. The bacterial load was significantly decreased after 1 week of Abx treatment and recovered at 2 weeks (Figure 2F), indicating that changes in the composition of gut microbiota were more important. Bacterial community richness and diversity analysis showed that Abx plus ADSCs treatment reconstructed a distinct bacteria profile as shown by alpha and beta diversity (Figure 2G-H). While ADSCs alone did not change the diabetic microbiota diversity significantly (Figure 2G-H). The bacterial taxa that exhibited the greatest changes following Abx treatment and ADSCs therapy were *Bifidobacterium*,* Streptophyta*, *Weissella*, *Lachnospiracea_incertae_sedis*,* Akkermansia* and* Acinetobacte,* which were reduced by co-housing (Figure 2I). These observations indicated that reducing the dysbiotic diabetic microbiota resistance could improve the ADSCs therapy; however, the underlying mechanism still needed to be explored.

### Abx treatment reduces translocation of the gut microbiota to the pancreas and improves insulin production

The immunomodulatory function is one of the important mechanisms of stem cell therapy. To understand the immunological implications of Abx plus ADSCs treatment, we analyzed the splenic T cell differentiation. Results showed that ADSCs treatment increased the frequency of splenic CD4+CD25+Poxp3+ Tregs as previously reported 20, while ADSCs plus Abx treatment failed to increase Tregs further compared with ADSCs group (Figure S2A). Interestingly short-chain fatty acids (SCFAs, Acetic Acid, Propanoic Acid and Butyric Acid), the metabolites produced by bacterial fermentation that are proposed to participate in Tregs induction were not different among the groups at 2 weeks (Figure S2B), suggesting that Tregs were enriched via other mechanisms in the combination treatment group.

**Figure 3:**
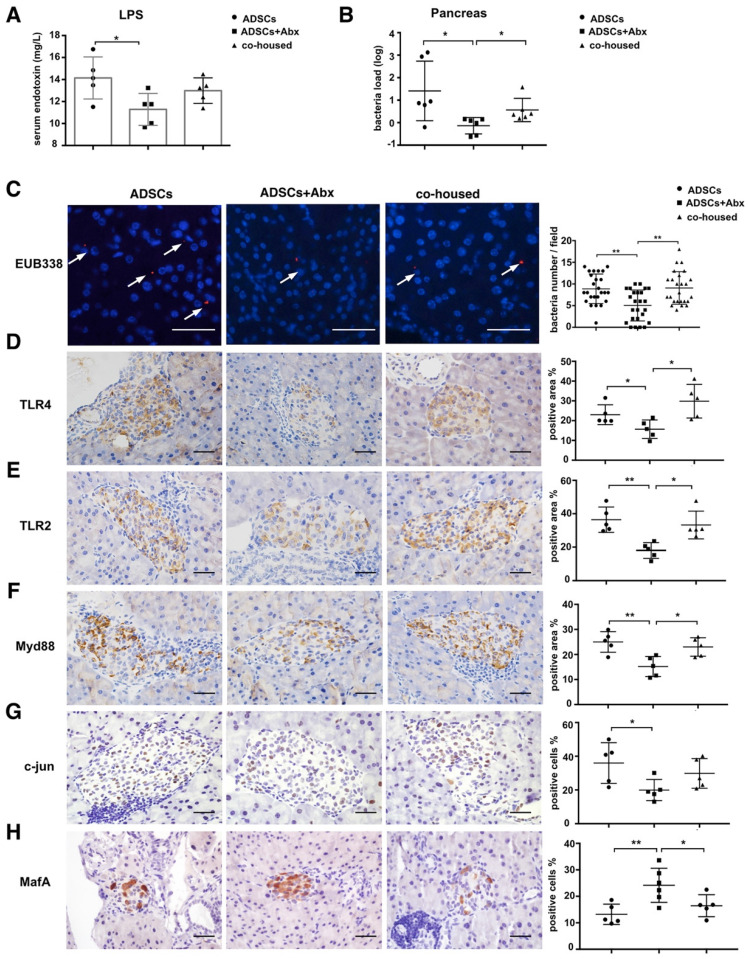
** ADSCs+Abx treatment reduces gut microbiota translocation to the pancreas and promotes insulin transcription.** (A) Serum LPS concentration, N=5. (B) Bacteria load in pancreas tissue detected by real-time PCR assay using a standard curve, N=6. (C) Bacteria detected in pancreas using in situ hybridization of general bacteria probe EUB338; cell nucleus stained with DAPI (blue) and bacteria stained with EUB338 (red), Scale bar: 40 μm. (D-H) Immunohistochemistry staining of TLR2, TLR4, Myd88, c-jun and MafA in pancreatic islets and quantified at right, N=5-6, Scale bar: 40 μm. Data is presented as Mean ± SEM. *P<0.05, **P<0.01.

To elucidate mechanisms through which Abx treatment and ADSCs therapy improved the diabetic condition, we examined changes in the intestinal barrier function and translocation of the gut microbiota to the pancreas 17. The serum lipopolysaccharide (LPS) levels were lower in the Abx plus ADSCs group compared to ADSCs alone (p=0.013, Figure 3A and p=0.0308 Figure S4E), and reduced bacterial levels in the serum (Figure S3A) and less bacterial presence in the pancreas were detected (Figure 3B). This observations were further confirmed by in situ hybridization staining using the eubacterial probe, EUB338 (Figure 3C). The negative control of EUB338 is shown in Figure S3D.

Since Toll like receptors (TLR) are known to recognize microbial pattern recognition receptors, we examined TLR2 and TLR4 expression in various groups. Both receptors were lower in the Abx plus ADSCs group compared with other groups (Figure 3D-E). Also, the expression of their downstream adaptor protein Myd88 was deceased in islets of the Abx plus ADSCs group compared to the group treated with ADSCs alone (Figure 3F). However, surprisingly, NF-κB, the key transcription factor related to inflammation downstream of Myd88, showed no significant activation in these three groups (Figure S3B). While another signaling factor, c-jun, downstream of Myd88 also had reduced expression with Abx and ADSCs treatment (Figure 3G). This was significant since MafA, which upregulates insulin expression and is negatively regulated by c-jun 24, increased in the Abx plus ADSCs group (Figure 3H). The results obtained by immunohistochemistry were confirmed at the mRNA level by real-time PCR (Figure S3C). Collectively, these results were consistent with the notion that Abx treatment combined with ADSCs transplantation reduced the translocation of the gut microbiota to the pancreas and enhanced insulin production.

### Abx treatment with ADSCs thickens the colonic inner mucus layer by promoting Mucin2 expression

**Figure 4:**
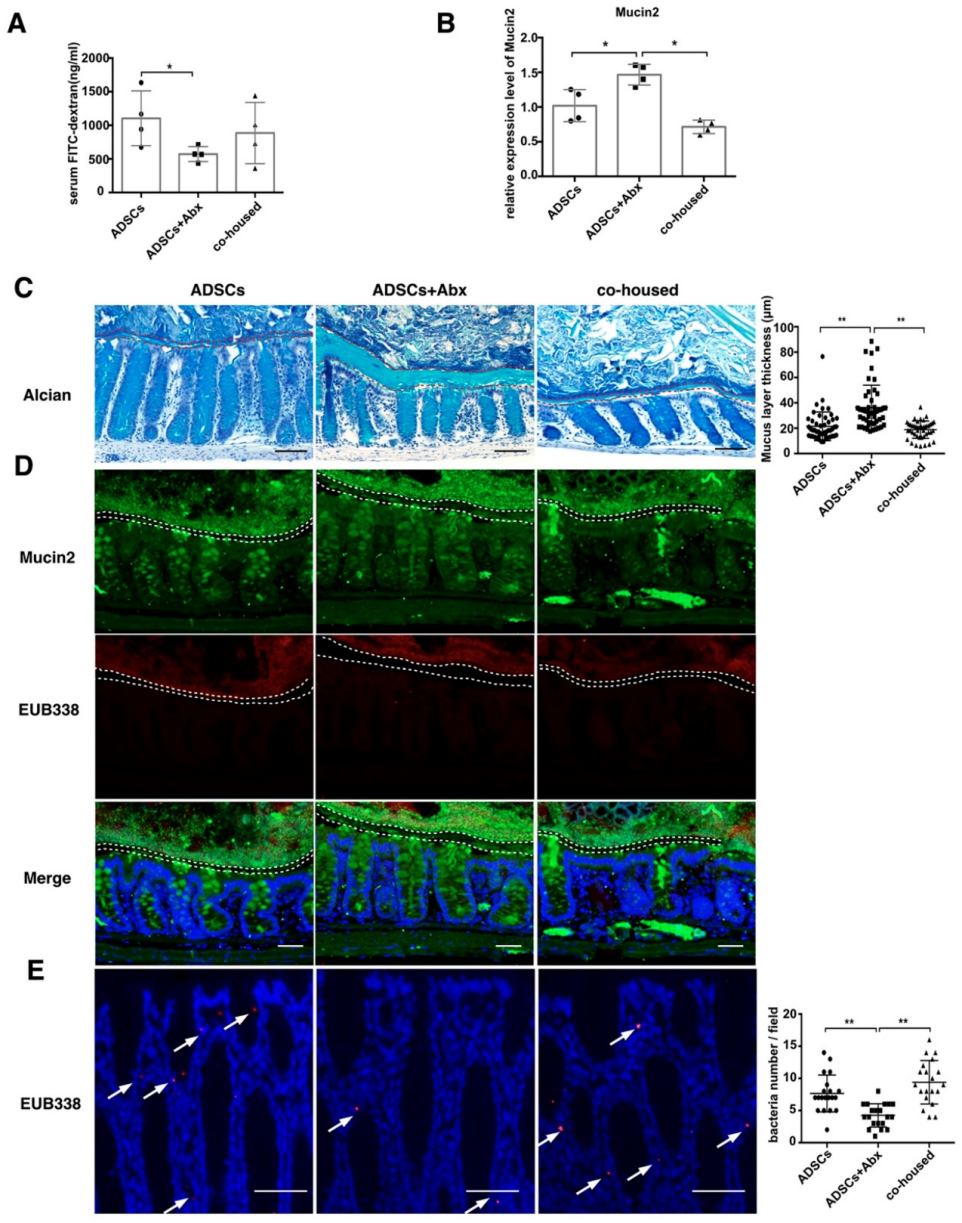
** ADSCs+Abx treatment decreases intestinal permeability by promoting Mucin2 expression and thickens colon mucus layer.** (A) Quantification of serum FITC-dextran concentration 4 hours after oral gavage, N=6. (B) Relative expression of Mucin2 in the mice colonic tissue. (C) Alcian staining of Carnoy's-fixed colon sections and quantification of mucus layer thickness (right), N=4-6, Scale bar:100 μm. (D) Dual immunofluorescence staining of Mucin2(green) and EUB338 (red) in Carnoy's-fixed colon sections, Scale bar: 40 μm. (E) Bacteria detected in colonic tissue using in situ hybridization of general bacteria probe EUB338, cell nucleus stained with DAPI (blue) and bacteria stained with EUB338 (red), Scale bar: 50 μm. Data is presented as Mean ± SEM. *P<0.05, **P<0.01.

To further investigate how the combined treatment could decrease bacterial translocation from the gut to the pancreas, we examined the barrier in the intestine. *In vivo* permeability assay using FITC-dextran revealed a decrease in permeability in mice subjected to the combined treatment compared to ADSCs therapy alone (p=0.0449, Figure 4A and p=0.031 Figure S4F).

To assess the integrity of the epithelium, we examined the tight junction protein Zonula occludens-1 (ZO-1) and found no significant differences between the three groups (Figure S4A). In contrast, the expression of Mucin2 was increased (Figure 4B) and the mucus layer thickness was higher in the combined treatment group versus treatment with ADSCs alone (Figure 4C). Expression of core 1 β1,3-galactosyltransferase (C1GalT1) and core 3 β1,3-N-acetylglucosaminyltransferase (C3GnT), related to mucin *O*-glycosylation, showed no significant difference (Figure S4B). The thickened mucus layer increased the distance between gut microbiota and epithelium (Figure 4D), and bacterial infiltration into the epithelium of the colon was also inhibited in the group with the combined treatment (Figure 4E). While ADSCs-treated mice had comparable permeability with PBS-treated mice such as mucus thickness and intestinal permeability (Figure S4C-D). The above results suggested that the depletion of diabetic gut microbiota was protective by up-regulating Mucin2 expression and inhibiting invasion of the intestinal epithelium.

### *B.breve* contributes to the thickening of the mucus layer induced by Abx treatment

We then attempted to identify whether specific microorganisms were altered by Abx plus ADSCs treatment that might contribute to the increased thickness of the inner mucus layer. The the overall bacterial load showed no significant difference at 2 weeks while the abundance of *Bifidobacterium* and *Akkermansia* was significantly increased in the Abx plus ADSCs group (Figure 2F and 2I). Interestingly, co-housing reversed this change. Previous studies reported that the abundance of *Bifidobacterium* and *Akkermansia* was negatively correlated with diabetes mellitus 25, 26. When the observation time extended to 4 weeks, the difference in blood glucose levels between ADSCs group and Abx plus ADSCs group reduced (Figure S5A), accompanied by the disappearance of *Bifidobacterium* abundance difference at 4 weeks (Figure S5B).

**Figure 5:**
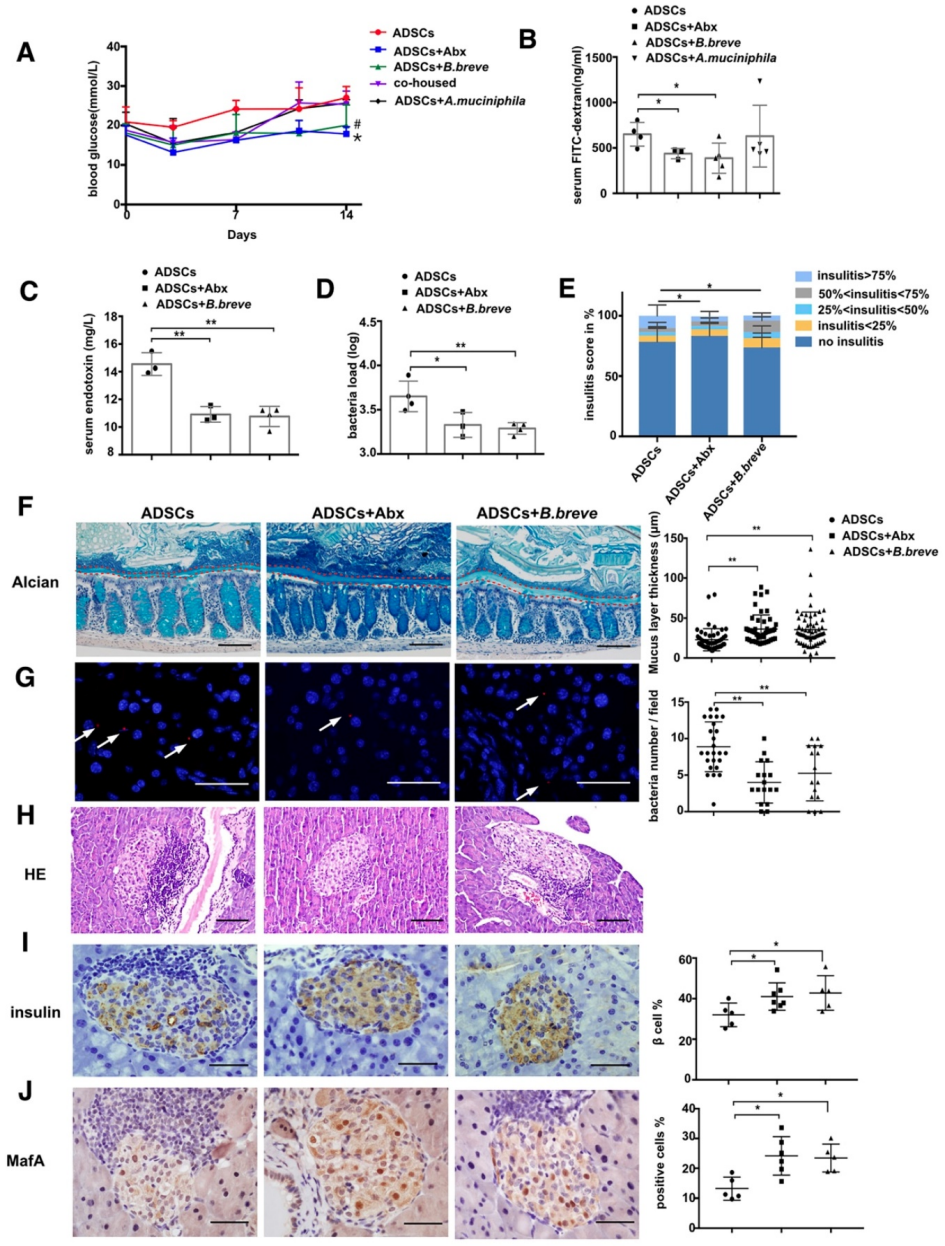
***B.breve* colonization during cohousing reduces intestinal permeability and promotes insulin expression.** (A) Blood glucose levels (mmol/L) was measured twice a week after ADSCs transplantation for 14 days, N=5. *P<0.05, co-housed vs ADSCs+Abx; ^#^P<0.05, co-housed vs co-housed+*B.breve*. (B) Quantification of serum FITC-dextran concentration 4 hours after oral gavage, N=3-5. (C) Serum LPS concentration, N=3-4. (D) Bacteria load in the pancreas tissue detected by real-time PCR assay using a standard curve, N=3-4. (E) Insulitis score in pancreatic islets. (F). Alcian staining of Carnoy's-fixed colon sections and quantification of mucus layer thickness (right), Scale bar:100 μm. (G) Bacteria detected in pancreas using in situ hybridization of general bacteria probe EUB338, cell nucleus stained with DAPI (blue) and bacteria stained with EUB338 (red), Scale bar: 40 μm. (H) H&E staining of pancreas at 2 week post ADSCs transplantation, Scale bar: 100 μm. (I-J) Immunohistochemistry staining of insulin and MafA in pancreatic islets and quantified at right, Scale bar: 40μm. Data is presented as Mean ± SEM. *P<0.05, **P<0.01.

To establish a causal relationship mice were given orally 1x10^9^ CFU of *B.breve* or *A.muciniphila* daily for one week and the successful colonization of *B.breve* and *A.muciniphila* in the gut were confirmed (Figure S6A-B). Gavage with *B.breve* decreased blood glucose levels compared to the ADSCs group on day 14 (p=0.0138, Figure 5A), indicating that the effect of *B.breve* was downstream of the Abx treatment. In other words, after Abx treatment, increased the population of *B.breve* could enhance the therapeutic effect of ADSCs transplantation. Furthermore, *in vivo* permeability assay showed decreased FITC-dextran leakage in ADSCs*+B. breve* group but not in the ADSCs+*A.muciniphila* group (ADSCs +*B.breve* 387.1 ± 74.44 ng/mL vs ADSCs 650.3 ± 65.37 ng/mL, p=0.0365; ADSCs+* A.muciniphila* 628.9 ± 152.1 ng/mL vs ADSCs 650.3 ± 65.37 ng/mL, p=0.909, Figure 5B).

Again, this result indicated that *B.breve*, and not *A.muciniphila*, was the key factor in Abx induced enhanced therapeutic effect of stem cell transplantation. Most importantly, the inner mucus layer showed increased thickness by application of *B.breve* compared to the treatment with ADSCs alone (p=0.0006, Figure 5F) and was accompanied by lower serum LPS levels (p=0.0013, Figure 5C) and reduced translocation of bacteria (Figure 5D and 5G). These changes were accompanied by a reduced propotion of islets with insulitis>75% (Figure 5H and 5E), an increase in MafA and an enhancement in insulin expression confirming that *B. breve* was a key bacterial component of the gut microbiota that was needed to protect against the development of hyperglycemia (Figure 5I-J).

### *B. breve* increases inner mucus layer thickness through TRPM7

**Figure 6:**
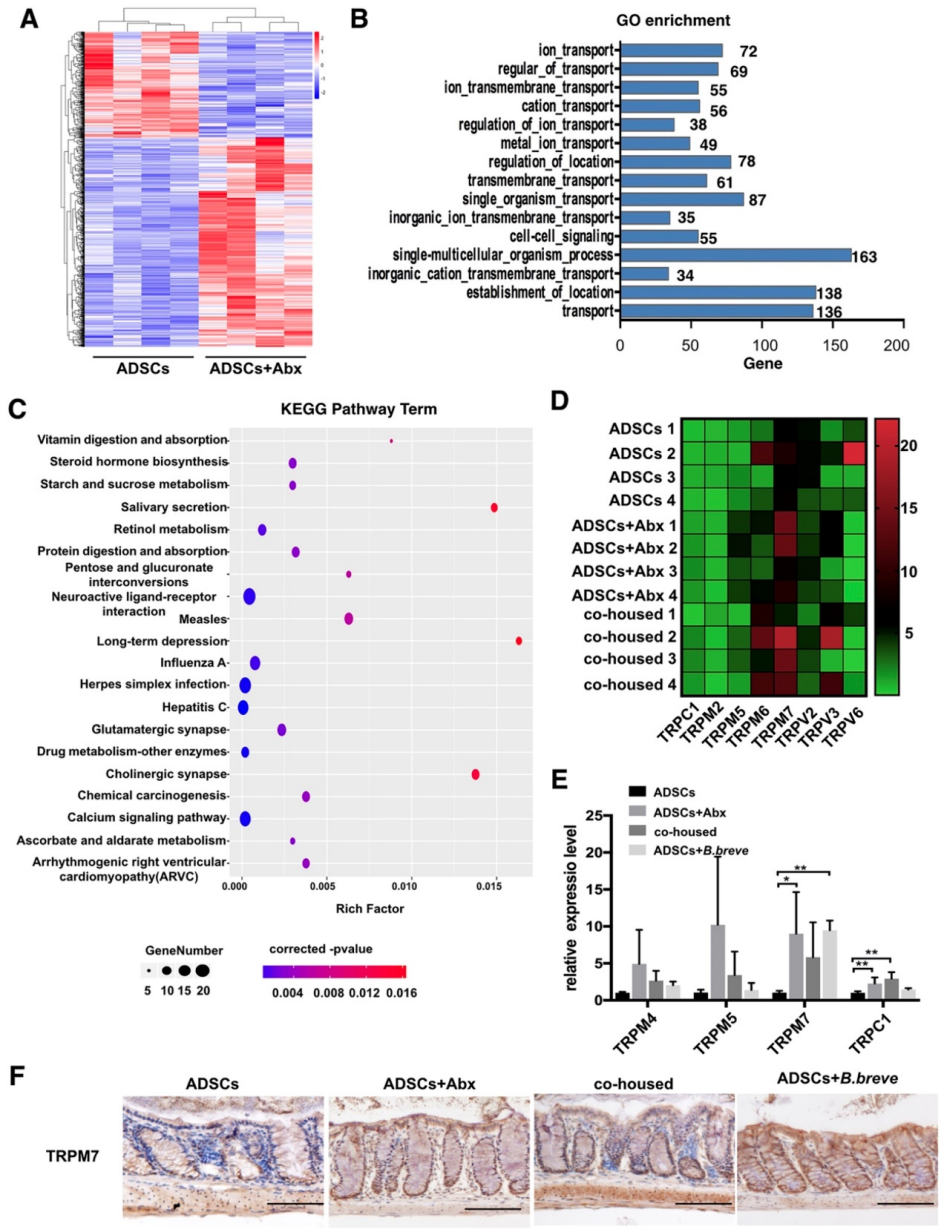
** TRPM7 is high expressed in colonic tissue after Abx or *B.breve* treatment.** (A) Gene expression profile of colonic tissue from ADSCs treated mice and Abx plus ADSCs treated mice, N=4. (B) GO enrichment analysis of biological processes of DEGs, the x-axis represents the number of DEGs, the y-axis represents the GO terms. (C) KEGG analysis of DEGs. (D) Heatmap of calcium signaling genes expression in colonic tissue from ADSCs treated mice, Abx plus ADSCs treated mice and cohoused mice. (E) Relative expression of TRPM4, TRPM5, TRPM7 and TRPC1 in mice colonic tissue. (F) Immunohistochemistry staining of TRPM7 in mice colonic tissue, Scale bar: 100 μm. Data is presented as Mean ± SEM. *P<0.05, **P<0.01.

To understand how Abx or *B.breve* promoted colon Mucin2 expression, we performed transcriptome sequencing of the colonic tissue from ADSCs-treated mice, Abx plus ADSCs-treated mice and co-housed mice. A different gene expression pattern was observed after the combined treatment in which 679 genes were upregulated and 344 genes were downregulated (Figure 6A). Subsequently, Gene Ontology (GO) enrichment analysis was performed to further understand the biological processes caused by these differentially expressed genes (DEGs), which showed prevalence of “ion transport” and “ion transmembrane transport” category (Figure 6B). After mapping these DEGs to the reference pathway in the Kyoto Encyclopedia of Genes and Genomes (KEGG) database, we found that the “Calcium signaling pathway” was highly enriched (Figure 6C).

Previous studies have reported that calcium signaling was involved in the secretion of mucin 27, 28, and transient receptor potential-melastatin-like 5 (TRPM5) channel was responsible for Mucin5AC secretion in human colonic cancer cell lines 29. We, therefore, analyzed the relative expression of other transient receptor potential (TRP) calcium channels. The results showed higher expression of TRPM7 in the colonic tissue of Abx plus ADSCs-treated mice compared to ADSCs-treated mice (Figure 6D). Real-time PCR also demonstrated high TRPM7 expression in both ADSCs plus Abx group and *B.breve*-treated group (Figure 6E). This observation was also confirmed by immunohistochemical staining of TRPM7 in the colonic tissue (Figure 6F). Thus, we hypothesized that Abx or *B.breve* might increase the increased mucin layer thickness through TRPM7.

**Figure 7:**
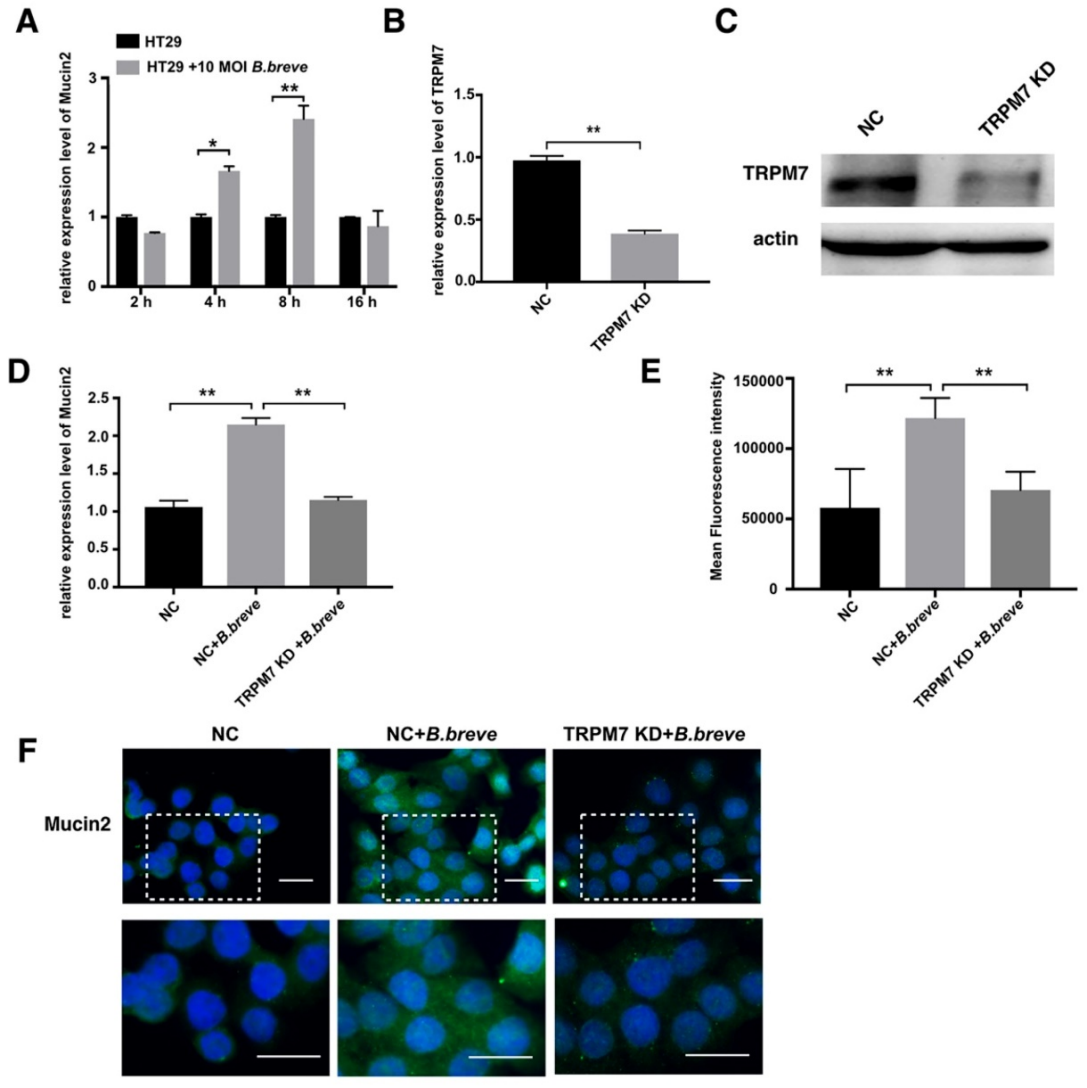
** TRPM7 played a pivotal role in *B.breve* mediated Mucin2 expression.** (A) Relative expression of Mucin2 in HT29 cells after *B.breve* incubation. (B-C) TRPM7 gene silencing efficiency detected by mRNA expression(B) and western blot (C). (D) Relative expression of Mucin2 in HT29 cells with or without TRPM7 knocked down after *B.breve* incubation. (E-F) Immunofluorescence staining of Mucin2 in HT29 cells with or without TRPM7 knocked down after *B.breve* incubation (F) and fluorescence intensity qualification (E). Scale bar: 20 μm. Data is presented as Mean ± SEM. *P<0.05, **P<0.01.

Next, *in vitro* studies using the human colonic adenocarcinoma cell line HT29 were performed to test this hypothesis. Cells were incubated with *B.breve* (MOI=10) for 2 h, 4 h, 8 h, and 16 h respectively, relative expression of Mucin2 was measured using real-time PCR. Treatment with* B.breve* promoted Mucin2 expression after 4 h and 8 h of incubation, and higher expression level of Mucin2 was achieved after 8 h of incubation (Figure 7A). To explore the role of TRPM7 in *B.breve* mediated Mucin2 expression, siRNA was applied to knock down TRPM7 in HT29 cells. The silencing efficiency of TRPM7 was confirmed by real-time PCR and Western blotting assay (Figure 7B-C). HT29 cells with or without TRPM7 knockdown were incubated with *B.breve* for 8h. As shown in Figure 7D, *B.breve* failed to induce higher Mucin2 expression in HT29 cells after TRPM7 was knocked down, and immunofluorescence staining also showed the same results (Figure 7E). These data further indicated that TRPM7 played a pivotal role in *B.breve*-mediated Mucin2 expression, which in turn verified our hypothesis.

## Discussion

About 78,000 youth are diagnosed with T1DM annually worldwide 30. ADSCs therapy is being actively pursued as a long-term treatment option for this disease but often suffers from long-term ineffectiveness 19, 31. In this study, we showed that treatment of mice with Abx during the application of ADSCs significantly improved the degree of hyperglycemia, while co-housing abolished this improvement. Co-housed mice were used to verify the role of gut microbiota for they acquire each other's gut microbiota because of their coprophagic property. Among the microbial species that was most affected was *Bifidobacterium*, which was increased while the overall bacterial load showed no significant difference. This specific change was one of the key factors in improving ADSCs outcomes by reducing bacterial translocation from the gut to the pancreas. Mechanistically, *Bifidobacterium* improved outcomes by enhancing the expression of a key gene, TRPM7, which stimulated the production of Mucin2 and subsequently enhanced the thickness of the colonic mucus later. Recent studies have shown that the movement of bacteria from the gut to the pancreas has a significant impact on the development of pancreatic cancer 32. Our studies provide important evidence that a similar movement of bacteria from the gut to the pancreas might limit the ability of stem cell therapy to treat T1DM effectively.

Increased intestinal permeability is a common characteristic in T1DM patients, pre-diabetic populations and animal models 15, 33. It is regulated by the intestinal epithelium and mucus layer, which are crucial in preventing the dissemination of bacteria from the gut lumen to the systemic circulation 34, 35. Our current study significantly extends previous work that the gut microbiota is important in the development of T1DM 36. It has been reported that oral gavage of cefotaxime and vancomycin can decrease intestinal permeability in Wistar rats 37. Our results that antibiotic could affect the mucus layer were supported by findings that metronidazole-treated rats have thickened colon mucus layer by increasing the *Bifidobacterium* genus, which has been shown to be positively correlated with Mucin-2 expression and mucus formation 27, 38, 39. However, the clinical benefits of this change were not established.

Besides, there is evidence in other studies that MSC therapy could reduce intestinal permeability, serum endotoxin level, and bacterial translocation in DSS-induced colitis model 40, 41, but we found comparable permeability between ADSCs and PBS groups. This might be due to the more severe intestinal inflammation in the colitis model leading to the recruitment of stem cells in the inflammatory site. The results also suggested that Abx and ADSCs work in different ways in our study. We found that antibiotic therapy alone did not improve the outcome. Rather, our results demonstrated that the principal effect of antibiotic treatment and *Bifidobacterium* treatment was a reduction in pancreatitis. This is significant since pancreatitis is thought to be an important factor in the success of mesenchymal stem cells therapy 20.

Gut bacteria translocation has been identified in a wide range of diseases 17, 42, 43. In our study, we found that intestinal permeability was decreased in the Abx plus ADSCs group, and real-time PCR assay and general bacteria probe staining showed a smaller bacterial load in the pancreas after Abx treatment. Furthermore, less intensive staining of microbial pattern recognition receptors TLR2 and TLR4 and their downstream adaptor protein Myd88 was also detected in the islets of Abx plus ADSCs-treated mice compared with ADSCs-treated mice. Both NF-κB and AP-1 are transcription factors downstream of Myd88 protein 44. NF-κB is required for a large number of inflammatory genes, including TNF-α, IL-1β, IL-6, IL-12p40 and cyclooxygenase-2 45. In our study, the NF-κB expression was weak and had no significant activation in ADSCs+Abx treatment. Other studies have reported that in NOD mice NF-κB was weakly activated despite progressive insulitis 46, suggesting its non-critical role of this factor. In contrast, the AP-1 family member c-jun was shown to be expressed in islet β cells 47. In diabetic db/db mice, the expression of c-jun in islets was upregulated compared to mice with normal blood glucose. The increase of the c-jun expression in both in MIN6 cells and in islets could suppress the expression of insulin transcription factor MafA and contribute to the decreased expression of insulin 24, 48. In our study, Abx plus ADSCs- treated mice showed fewer c-jun positive cells and more MafA positive cells in islets. This was also consistent with intensive insulin staining in the Abx plus ADSCs group.

Many studies have reported the effects of antibiotics on the development of T1DM, however, the results are controversial. Many studies described that antibiotics accelerated T1DM in NOD mice 3, 12, 49, while other studies found that antibiotics are found to be protective. Hansen et al. reported that mice receiving vancomycin from birth until weaning (day 28) showed reduced diabetes incidence 25. Brugman et al. also found that treatment with antibiotics (sulphamethoxazole, trimethoprim, colistine sulphate) reduced diabetes incidence and delayed its onset by 30 days 50. In addition, female and male NOD mice also responded differently to the same antibiotics 49. More importantly, the gut microbiota is dynamic balanced 5, before and after the onset of diabetes the microbiota are different 51. In all the studies mentioned above, antibiotics were treated before the onset of diabetes in NOD mice, which perturbed the healthy gut microbiota. This might explain why the use of antibiotics before the onset of the disease increases the incidence of diabetes in most studies.Also antibiotic treatment yields different results before and after the onset of the disease 52. Thus, antibiotic type, dose, timing and animal model are all relevant variables that might influence effects. In our study, antibiotics were used to deplete the diabetic gut microbiota after the onset of the T1DM. We found that the use of antibiotics reduced the intestinal permeability and translocation of gut microbiota, which were consistent with other studies 17, 53.

In summary, we found that diabetic gut microbiota could abolish the therapeutic effects of MSC therapy, while depletion of this “gut microbiota resistance” by antibiotics enhanced therapeutic effect of MSC therapy. However it remains to be determined which ectopic bacterial colonization affects the function of the pancreas. We believe our findings provide the rationale for a new approach to MSC therapy by modulating the gut microbiome. For patients with dysbiotic gut microbiota or patients who do not respond to MSC therapy, the regulation of gut microbiota might help achieve a better therapeutic effect and extend the prospect of its clinical application in the future, providing benefit to more patients. In this respect, the availability of only animal model data is a serious limitation, and clinical trails to test promising pre-clinical data are highly desirable.

## Methods

### Mice

7-8 weeks of age female NOD/Ltj mice were purchased from Peking University Health Science Center Experimental Animal Science Center (Beijing, China) and reared under specific-pathogen-free conditions at a cycle of 12:12 light/dark with constant temperature and humidity. All animal procedures were approved by Ethics Committee of the Peking University Health Science Center (approval number: LA2019190).

### Cell Culture

Human adipo-derived stem cells (ADSCs) were isolated from healthy donors as previously described 54. All procedures were approved by the Ethics Committee of Peking University (PKUSSIRB-201948106). ADSCs were culture in alpha-MEM medium (Gibco, Grand Island, NY, USA) with 10%FBS at 37°C. The cells at passage 2-5 were used for transplantation.

Human colonic adenocarcinoma cell line (provided by Professor Yixiang Wang, Peking University School and Hospital of Stomatology, Beijing, China) was maintained in DMEM (Gibco, USA) medium with 10%FBS at 37°C.

### Diabetes animal model and treatment

Individual NOD/Ltj mice were injected intraperitoneally with 45 mg/kg streptozocin (STZ, Sigma-Aldrich, St. Louis, MO, USA) for four consecutive days to accelerate diabetes development and progression. Individual mice with blood glucose levels >13.9 mmol/L for three consecutive days were diagnosed as diabetes. We exclude the bias, including mice failed to develop diabetes and euthanized mice whose blood glucose exceeded the glucometer range. For treatment, one group of diabetes mice were injected with 1.0 × 10^6^ ADSCs resuspended in PBS solution (100μl) intravenously twice at an interval of one week (ADSCs group). The other group of mice were injected with PBS as negative control (PBS group). To test the role of diabetic gut microbiota in ADSCs treatment, we co-housed the PBS-treated mice and ADSCs-treated mice together (PBS/ADSCs co-housed group). Co-housed mice acquire each other's gut microbiota because they are coprophagic; therefore through co-housing ADSCs mice could receive the gut microbiota from PBS-treated diabetic mice.

### Antibiotic treatment

To deplete gut microbiota, mice were treated with broad spectrum antibiotic cocktail (Abx, ampicillin (1 mg/ml), metronidazole (1 mg/ml), neomycin (1 mg/ml) and vancomycin (0.5 mg/ml)) in drinking water for 1w simultaneously with the first time of ADSCs injection (ADSCs+Abx group). To investigate the role of gut microbiota in ADSCs transplantation, we recolonized ADSCs+Abx treated mice with microbiota by cohousing with ADSCs treated mice, which was considered as co-housed group. To eliminate the effects of the antibiotics by themselves, we treated one group of mice with Abx alone for 1 week. Besides, we divided the components in Abx into three groups, ampicillin and vancomycin group (Amp+Van), neomycin group (Neo) and metronidazole group (Met), and treated mice with these three groups of antibiotics with ADSCs tansplantation respectively for 1 week.

### Serum Insulin and LPS Levels

Mice serum insulin concentration were assessed by mouse insulin Elisa Kit (Mercodia AB, Uppsala, Sweden) after a 6-hour fast according to the manufacturer's instructions. And mouse LPS concentrations were assessed by LPS Elisa Kit (Jingmei, Jiangsu, China) according to the manufacturer's instructions.

### Histological analysis of the pancreas

Pancreas were fixed in 10% Neutral Buffered-formalin and embedded in paraffin. A 4μm sections were cut using a microtome (Leica Microsystems), and three sections with a 120μm gap were chosen and measured for each pancreas. These sections were deparaffinized, rehydrated and stained with hematoxylin and eosin (H&E). Five to seven mice in each group participated in the scoring, and each mouse had three tissue slices. Insulitis was evaluated on a 0-4 scale (0, no insulitis; 1, leukocyte infiltration <25%; 2, leukocyte infiltration>25%, but <50%; 3, leukocyte infiltration >50%, but <75%; 4, leukocyte infiltration >75% and β-cell destruction)^3^. Insulitis scoring was performed by two researchers, blinded to each other, independently reviewed the slide scans.

### Immunohistochemistry and Immunofluorescence Staining

For immunohistochemistry staining, sections were deparaffinized, rehydrated and treated with 3% H_2_O_2_ in methanol for 20 min to inactivate endogenous peroxidase activity. Then sections were subjected to antigen retrieval and treated with serum albumin (BSA) for 1 h. Subsequently, the sections were incubated with rabbit anti-insulin antibodies (1:100; Proteintech, Wuhan, China), rabbit anti-TLR2 antibodies (1:100; Abcam, Cambridge, MA,USA), rabbit anti-TLR4 antibodies (1:100; Santa Cruz Biotech, California, USA), rabbit anti-Myd88 antibody (1:100; Proteintech, China), rabbit anti-NF-κB antibody (1:100; Proteintech, China), rabbit anti-c-jun antibody (1:100; Proteintech, China), rabbit anti-MafA antibody (1:100; Abcam, USA), rabbit anti-ZO-1 antibody (1:50; Proteintech, China) overnight at 4 °C. For immunohistochemistry staining the bound antibodies were detected with HRP-conjugated secondary antibody and visualized with DAB (Zhongshan Biosciences Inc., Beijing, China). For immunofluorescence staining, slices were incubated with FITC-labelled secondary antibody (Zhongshan Biosciences Inc., China) for 1h at RT and mounted with 4',6-diamidino-2-phenylindole (DAPI, Zhongshan Biosciences Inc., China). After being imaged, the levels of insulin, TLR2, TLR4, Myd88, NF-κB, c-jun and MafA staining in individual images were measured as the ratios of positively staining IOD in islets to total islets areas imaged. For each marker the sample size is as follows: for TLR2 staining: ADSCs group: 5 mice; Abx plus ADSCs group:5 mice; co-housed group:5 mice; for TLR4 staining: ADSCs group: 5 mice; Abx plus ADSCs group:5 mice; co-housed group:5 mice; for Myd88 staining: ADSCs group: 5 mice; Abx plus ADSCs group:5 mice; co-housed group:5 mice; for c-jun staining: ADSCs group: 5 mice; Abx plus ADSCs group:5 mice; co-housed group:5 mice; for MafA staining: ADSCs group: 5 mice; Abx plus ADSCs group:6 mice; co-housed group:5 mice; for insulin staining: PBS group: 6 mice; ADSCs group: 5 mice; Abx plus ADSCs group:7 mice; co-housed group:6 mice.

### Fluorescent in situ hybridization (FISH)

We used FISH analysis to identify and localize bacteria in the pancreas and colon. The following probe (EUB338-FAM, GCT GCC TCC CGT AGG AGT) was used to detect general bacteria; the probe was diluted to a working concentration of 5ng/μl with a hybridization buffer (100 mM Tris-HCl, 0.9 M Nacl, 0.1% SDS, 20% Formamide, pH 7.5). A non-Eub338-FAM (ACTCCTACGGGAGGCAGC) probe was used as a negative control (Figure S2D). Formalin or Carnoy's fixed paraffin-embedded tissue sections (4μm) were deparaffinized, rehydrated, and after air dried and then slides were incubated with EUB338 or negative control at 56°C overnight. Samples were then washed in washing buffer (100 mM Tris-HCl, 0.9 M Nacl, pH 7.5) at 46°C for 20 min. Slides were rinsed in distilled water, air-dried, and mounted with DAPI. Hybridized sections were examined on an Olympus BX51 (Olympus America, Melville, NY) epifluorescence microscope and images captured with an Olympus DP-70 camera. For dual staining of EUB338 and Mucin2, we used two adjacent slides to stain EUB338 and Mucin2 respectively and merge the image with Olympus BX51.

### Histological staining of mucins and calculation of mucus thickness

Carnoy's fixed colon sections were cut (4μm), deparaffinized and rehydrated. Sections were then immersed with Alcian blue (AB) pH 2.5 (Solarbio, Beijing, China), which stains acidic mucins a light blue, for 15 min and then thoroughly rinsed in tap water. Sections were counterstained with Hematoxylin for 3 min, dehydrated and mounted with neutral gum and imaged.

For determination of mucus thickness using Alcian blue tissue sections, 10-15 measurements were taken from each animal colon. Each measurement was taken perpendicularly to the upper and lower borders of the inner mucus layer, after which the mean absolute distances were determined. ImageJ software was used for data acquisition.

### In Vivo Permeability Assay

Intestinal permeability was determined by FITC- dextran assay. Briefly, FITC-conjugated dextran (FITC-dextran; molecular mass, 4kDa; Sigma-Aldrich, USA) was dissolved in PBS to a concentration of 80 mg/ml. Mice were fasted for 4 hours prior to oral gavage with 150μl FITC-dextran. After 4 h, blood was collected and centrifuged at 1,000 x g for 30 min at 4°C. Serum was collected and the concentration of fluorescein was quantified at excitation wavelength of 485 nm and emission wavelength of 535 nm using serially diluted samples of the FITC-dextran marker as standard.

### Tissue and cell RNA extraction and PCR amplification

Total RNA was isolated from tissue with TRIZOL reagent (Invitrogen, California, USA), and 2μg RNA were reverse-transcribed into cDNA using the SuperScript First-Strand Synthesis System (Invitrogen, USA), according to manufacturer's instructions. These reactions were performed in a 20μL reaction mixture with ABI 7500 real-time PCR system (Applied Bioscience, PerkinElmer, Foster City, CA). The expression of TLR2, TLR4, Myd88, c-jun, MafA, Mucin2, c3gnt1, cigalt1 were determined and normalized with mice β-actin using ΔΔCt method. All primers used in these experiments are described in **Table 1**.

### Bacteria culture of blood

Blood samples were collected by cardiac puncture, 100μl blood was plated on tryptic soy agar medium (Sigma, USA) at 37°C. The bacterial load was quantified by counting CFU on each plate after overnight incubation.

### Tissue and fecal DNA extraction, quantification and 16s rRNA sequencing

Fresh stool samples were collected using sterile centrifuges. Approximately 0.04g of stool and 0.1g pancreas tissue were used to extract microbiome by a DNeasy kit (QIAGEN, Valencia, California, USA) as described by the manufacturer. To quantify the total bacteria load, universal 16S rRNA gene primers (341F: ACTCCTACGGGAGGCAGCAG, 806R: GGA CTA CHV GGG TWT CTA AT) targeting the V3-4 region were used for quantitative real-time PCR (real-time PCR) assay. Standard curves were constructed using PCR product of the 16S rRNA gene of E. coli by serial diluting.

For fecal DNA 16s rRNA sequencing, the V3-V4 hypervariable regions of the 16S rRNA gene were subjected to high-throughput sequencing by Shanghai Realbio Technology, Co., Ltd (Shanghai, China) using the Illumina Miseq PE250/ Hiseq PE250 sequencing platform (Illumina, Inc., CA, USA). Data were analyzed using QIIME software with Greengenes database (2013 updated version). The sample size of mice involved in 16s rRNA sequencing is as follows: PBS group: 7 mice; ADSCs group: 9 mice; PBS/ADSCs cohoused group: 11 mice; Abx plus ADSCs group:12 mice; co-housed group:7 mice. The raw sequencing data of this study are available in the NCBI Sequence Read Archive with accession number SRP229799.

### B.breve and A.muciniphila Supplementation

*Bifidobacterium Breve* (NO.1.3001, provided by Dr. Lin Wang and Prof. Chuanbin Guo, Peking University School and Hospital of Stomatology, Beijing, China) and *Akkermansia muciniphila* (ATCC BAA-835, provided by Prof. Lanjuan Li, Zhejiang University, Hangzhou, China) were cultured anaerobically in MRS and mucin-based BHI medium separately at 37℃. After receiving ADSCs transplantation and Abx treatment, mice were given 1x10^9^ CFU *B.breve* and *A.muciniphila* orally every day for one week during cohousing. And these two group were referred as ADSCs+* B.breve* and ADSCs +*Akkermansia* group respectively.

To test colonization of *B.breve* and *A.muciniphila*, mice stool was collected and frozen at -80℃. DNA was extracted as previous described. Genomes extracted directly from cultured *B.breve* and *A.muciniphila* served as control groups respectively. The V3-V4 region of 16S rRNA gene was amplified by PCR using primers *B.breve* F 5'-CCGGATGCTCCATCACAC-3' and *B.breve* R 5'-ACAAAGTGCCTTGCTCCCT-3'; *A.muciniphila* F 5'-CAGCACGTGAAGGTGGGGAC-3' and *A.muciniphila* R 5'-CCTTGCGGTTGGCTTCAGAT-3'. Goaq Green Master Mix (Promega, USA) contained GoTaq DNA Polymerase, dNTPs, MgCl2 and reaction buffers at optimal concentrations, and 50ng gDNA template. Cycling conditions were 95°C for 2min, and 35 cycles of 95°C for 30sec, 60°C for 30sec, 72°C for 1min, and 72°C for 10min using Eppendorf Mastercycler nexus PCR machine. 10μl PCR products were verified by electrophoresis in 1.5% (w/v) agarose gels (Biowest, Barcelona, Spain) at the constant voltage of 120 V for 40 min in 1× TAE buffer.

### Transcriptome sequencing

Total RNA from colonic tissue were extracted as previous described. Briefly, a total amount of 1 µg RNA per sample was used as input material for the RNA-seq libraries preparation using NEBNext® Ultra™ RNA Library Prep Kit for Illumina® (NEB, USA) following manufacturer's recommendations. The clustering of the index-coded samples was performed on a cBot Cluster Generation System using TruSeq PE Cluster Kit v3-cBot-HS (Illumia) according to the manufacturer's instructions. After cluster generation, the library preparations were sequenced on an Illumina NovaSeq platform and 150 bp paired-end reads were generated. The resulting FASTQ files were analyzed via FastQC to ensure sufficient data quality.

### Effect of *B.breve* on Mucin2 expression in HT29 cells

HT29 cells were plated in six-well plate in antibiotic-free DMEM medium. After the cells reached about 60-70% confluence, *B.breve*(MOI=10) were added to cells and total RNA were extracted after 2 h, 4 h, 8 h and 16 h incubation.

### *In vitro* siRNA interference assays

HT29 cells were plated in six-well plate in DMEM medium. The transfection medium was made with opti-MEM (Invitrogen, Carlsbad, CA, USA) combined with TGF-β1-specific or scrambled siRNA (RiboBio Co. Ltd., Guangzhou, Guangdong) and Lipofectamine RNAi-max (Invitrogen, Carlsbad, CA, USA) according to the instructions. Total RNA was extracted after 24 h of transfection and total protein was extracted after 48 h of transfection.

### Western Blot

Total proteins were extracted using RIPA lysis buffer. Proteins were separated on SDS-PAGE, and transferred to nitrocellulose filter membranes, which were probed with the following antibodies overnight at 4°C: anti-TRPM7 (1:1000; Abcam, Cambridge, MA, USA), anti-β-actin antibodies. Immunocomplexes were detected with an enhanced chemiluminescence blotting kit (Applygen Technology Inc., Beijing, China).

### SCFAs measurement

Mice serum samples were processed for short fatty acid (acetic acid, propanoic acid and butyric acid) analysis using gas chromatography.

### Flow Cytometric Analysis

Splenic mononuclear cells were isolated and the frequency of CD4^+^CD25^+^Foxp3^+^ Tregs in individual mice was determined by flow cytometry. Furthermore, splenic mononuclear cells were stained in duplicate with FITC-anti-CD4 (eBioscience, San Diego, USA) and APC-anti-CD25 (eBioscience, USA), fixed, permeabilized and stained intracellularly stained with PE-anti-FoxP3 (eBioscience, USA). The frequency of CD4+CD25+Foxp3+ Tregs in total CD4+ T cells were analyzed with the FlowJo software.

### Statistical analysis

The results were presented as the mean ± SEM. Differences between two unpaired groups were compared by Student's t test. Statistical variations between three or more groups within an experiment were analyzed by one-way ANOVA, followed by Tukey's post-test, using the Prism 6.0 statistical program (GraphPad). P < 0.05 was considered statistically significant.

## Supplementary Material

Supplementary figures and tables.

## Figures and Tables

**Table 1:** The primers used for real-time PCR

Name	Primer	Sequence (5'-3')
m-TLR2	Forward	TCTGGGCAGTCTTGA ACATTT
	Reverse	AGAGTCAGGTGATGGATGTCG
m-TLR4	Forward	CAAGGGATAAGA ACGCTGAGA
	Reverse	GCA ATGTCTCTGGCAGGTGTA
m-Myd88	Forward	ACCTGTGTCTGGTCCATTGCCA
	Reverse	GCTGAGTGCAAACTTGGTCTGG
m-c-jun	Forward	TCCCCTATCGACAT GGAGTC
	Reverse	TTTTGCGCTTTCAAGGTTTT
m-MafA	Forward	TTCAGCAAGGAGGAGGTCAT
	Reverse	CCGCCAACTTCTCGTATTTC
m- Mucin2	Reverse	AGGGCTCGGAACTCCAGAAA
	Reverse	CCAGGGAATCGGTAGACATCG
m-C3GnT	Forward	GGGCGTCGAAGAGTCAAGTT
	Reverse	TTTCGGGATTATTACTCCTCCCT
m-C1GalT1	Forward	TGGAATTACAACTATTATCCTCCCATA
m-TRPM4	Reverse	CAACATAGTGAAAAGAAACTGCGATA
	Forward	ACCGAGTGGAACAGTGATGAG
	Reverse	CCAAGAGCGGGTAACGAGAC
m-TRPM5	Forward	CCAGCATAAGCGACAACATCT
	Reverse	GAGCATACAGTAGTTGGCCTG
m-TRPM7	Forward	AGGATGTCAGATTTGTCAGCAAC
	Reverse	CCTGGTTAAAGTGTTCACCCAA
m-TRPC1	Forward	GATGTGCTTGGGAGAAATGCT
	Reverse	ACTGACAACCGTAGTCCAAAAG
m-actin	Forward	GTGACGTTGACATCCGTAAAGA
	Reverse	GCCGGACTCATCGTACTCC
h-actin	Forward	CATGTACGTTGCTATCCAGGC
	Reverse	CTCCTTAATGTCACGCACGAT
h-Mucin2	Forward	GAGGGCAGAACCCGAAACC
	Reverse	GGCGAAGTTGTAGTCGCAGAG
h-TRPM7	Forward	ACTGGAGGAGTAAACACAGGT
	Reverse	TGGAGCTATTCCGATAGTGCAA

## Abbreviations

ADSCs: Adipose-derived stem cells
C1GalT1: core 1 β1,3-galactosyltransferase
C3GnT: core 3 β1,3-N-acetylglucosaminyltransferase
DEG: differentially expressed genes
GO: gene ontology
HE: hematoxylin and eosin
KEGG: Kyoto Encyclopedia of Genes and Genomes
LPS: lipopolysaccharide
MafA: v-maf musculoaponeurotic fibrosarcoma oncogene homologue A
MOI: multiplicity of infection
MSC: mesenchymal stem cell
Myd88: myeloid differentiation primary response 88
NOD: non-obese diabetic mice
NF-κB: nuclear factor kappa-light-chain-enhancer of activated B cells
PBS: phosphate-buffered saline
si RNA: small interfering RNA
STZ: streptozotocin
SCFA: short-chain fatty acids
T1DM: type 1 diabetes mellitus
TLR: Toll like receptor
TRP: transient receptor potential
TRPM5: transient receptor potential-melastatin-like 5
TRPM7: transient receptor potential-melastatin-like 7
ZO-1: zonula occludens-1
