# Supplementary material for: Depletion of the diabetic gut microbiota resistance enhances stem cells therapy in type 1 diabetes mellitus
Source: Theranostics. 2020 May 17;10(14):6500–16. doi: 10.7150/thno.44113 (PMC7255019; doi:10.7150/thno.44113)
Supplement: Supplementary file 1 — Supplementary figures and tables. [file thnov10p6500s1.pdf]

Supplementary Materials

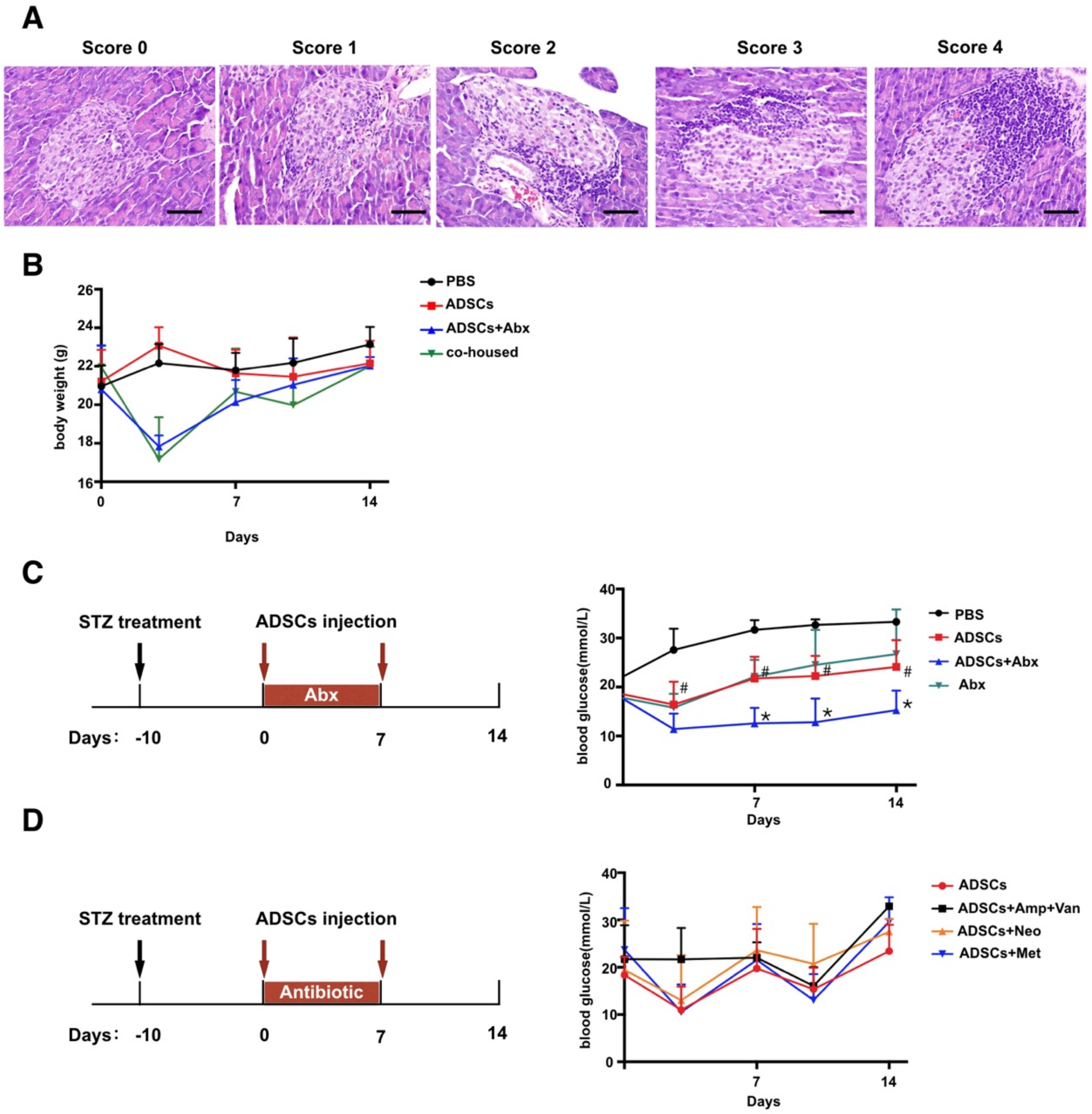

Figure S1. Abx alone or individual components of Abx combined with ADSCs did not further reduced blood glucose levels. (A) Degree of insulitis as scored on representative H&E stained islets (range 0–4). Scale bars: 50 µm. (B) Body weight (g) was measured twice a week after ADSCs transplantation for 14 days. (C) Blood glucose levels (mmol/L) of PBS, ADSCs, ADSCs+Abx and Abx group were measured twice a week after ADSCs transplantation for 14 days (N=5-8). *P<0.05, ADSCs vs ADSCs+Abx; $^{\#}$P<0.05, PBS vs ADSCs. (D) Blood glucose levels (mmol/L) of ADSCs, ADSCs+Amp+Van, ADSCs+Neo, ADSCs+Met group were measured

twice a week after ADSCs transplantation for 14 days (N=4-5). Data is presented as Mean ± SEM. *P<0.05, **P<0.01.

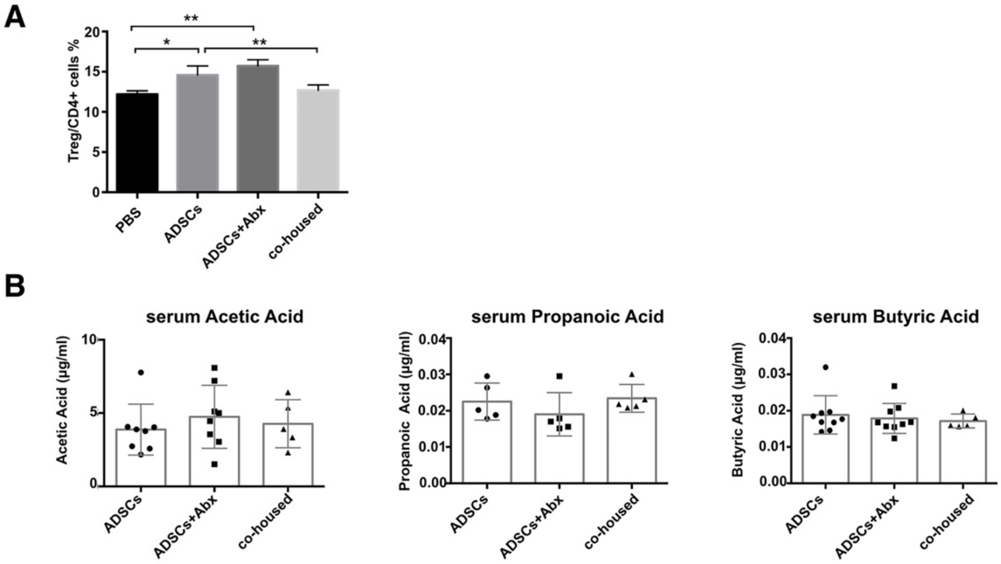

Figure S2. ADSCs+Abx treatment showed no effect on splenic Tregs frequency and serum SCFA concentration. (A)The frequency of splenic Tregs in total CD4+ T cells, N=5. (B) Concentration of Acetic Acid, Propanoic Acid and Butyric Acid level in mice serum, N=5-9. Data is presented as Mean ± SEM. *P<0.05, **P<0.01.

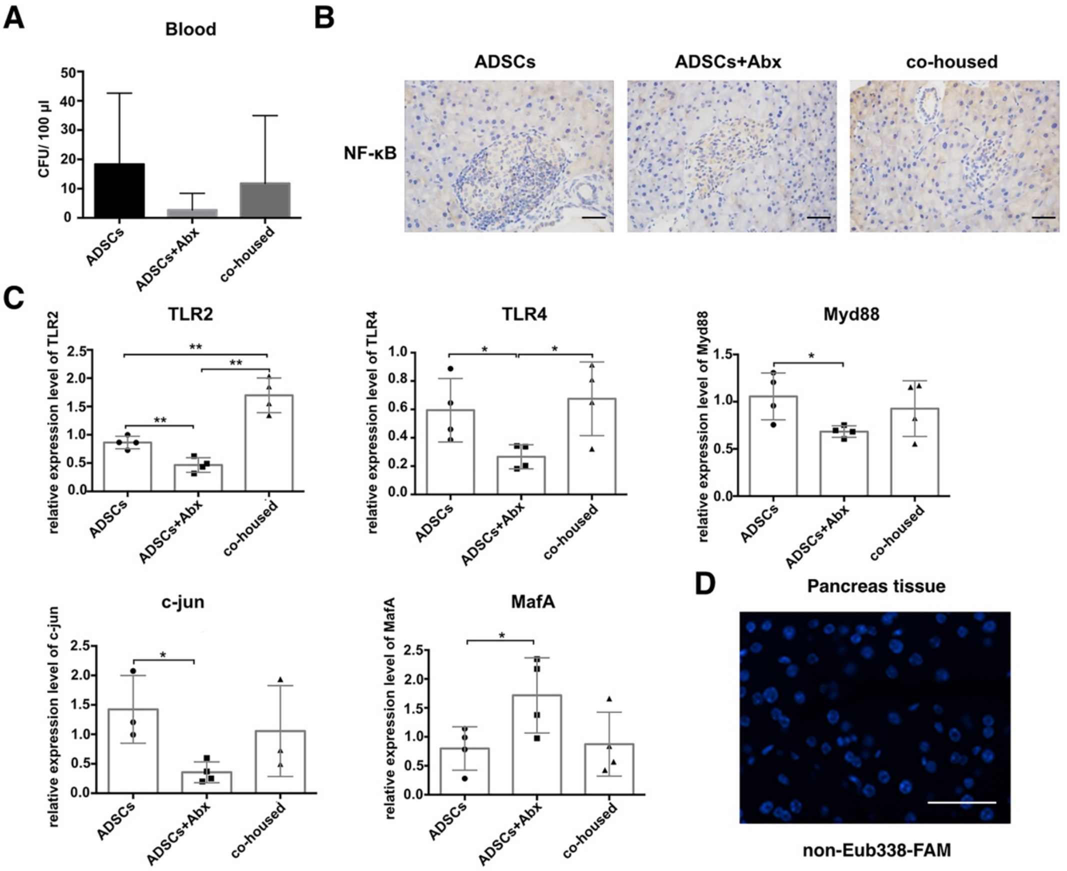

Figure S3. c-jun, not NF-κB, is required for ADSCs+Abx treatment induced insulin production improvement. (A) Blood bacteria loads in ADSCs, ADSCs+Abx and co-housed mice. (B)Immunohistochemistry staining of NF-κB in pancreatic islets, Scale bar: 40 μm. (C) Relative expression of TLR2, TLR4, Myd88, c-jun and MafA in pancreatic tissue, N=4. (D) Negative control of Fish assay, staining the pancreatic tissue with non-Eub338-FAM, Scale bar: 40 μm. Data is presented as Mean ± SEM. *P<0.05, **P<0.01.

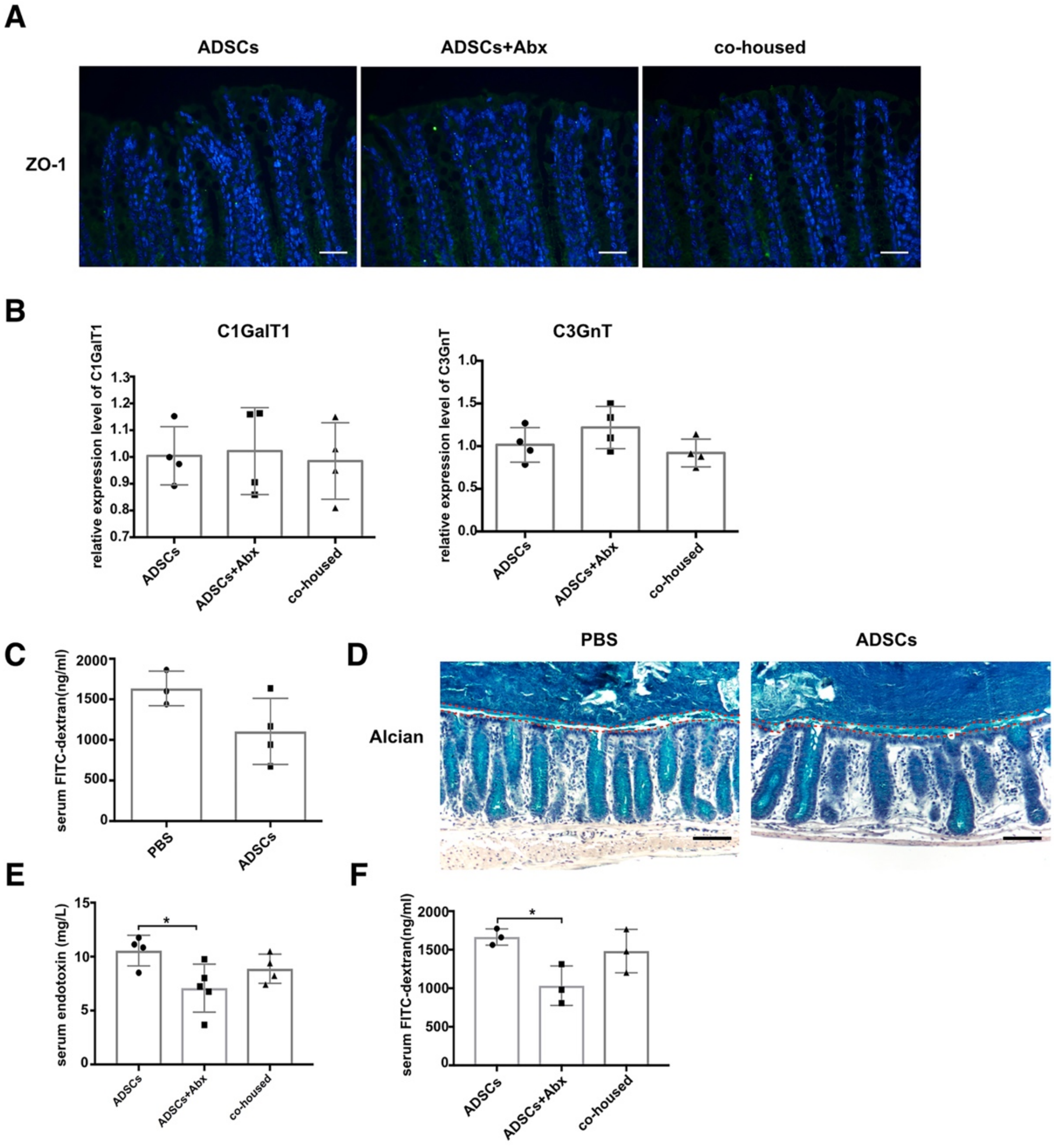

Figure S4. ADSCs+Abx treatment did not affect intestinal epithelium barrier and mucin glycosylation. (A) Immunofluorescence staining of ZO-1, Scale bar: 40 μm. (B) Relative expression of C1GalT and C3GnT in mice colonic tissue, N=4. (C) Serum FITC-dextran concentration of PBS and ADSCs group , N=3-4. (D) Alcian staining of Carnoy's-fixed colon sections, N=4-5, Scale bar: 100 μm. (E) Serum LPS concentration, N=4-5. (F) Quantification of serum FITC-dextran concentration 4 hours after oral gavage, N=3. Data is presented as Mean ± SEM. *P<0.05, **P<0.01.

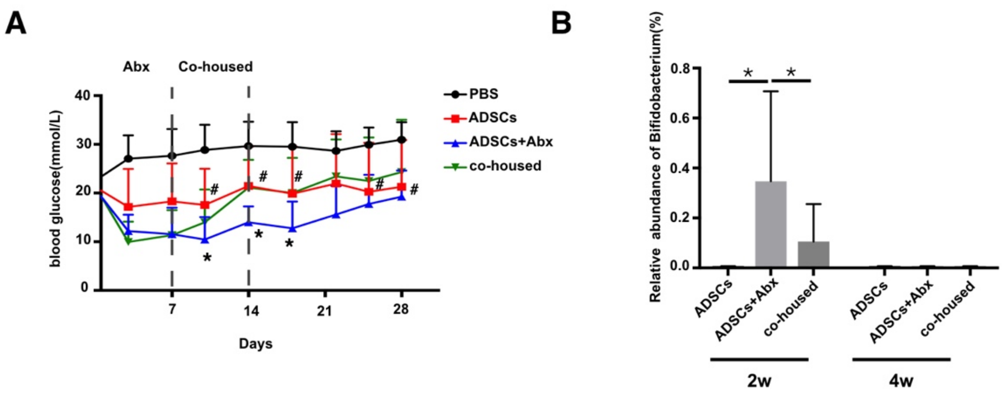

Figure S5. blood glucose levels and *Bifidobacterium* abundance showed no significant difference between ADSCs+Abx group and ADSC group at 4 weeks. (A) Blood glucose levels (mmol/L) were measured twice a week after ADSCs transplantation for 28 days (N=6-8). *P<0.05, ADSCs vs ADSCs+Abx ; $^{\#}$P<0.05, PBS vs ADSCs. (B) Relative abundance of *Bifidobacterium* at 2 weeks and 4 weeks. Data is presented as Mean ± SEM. *P<0.05, **P<0.01.

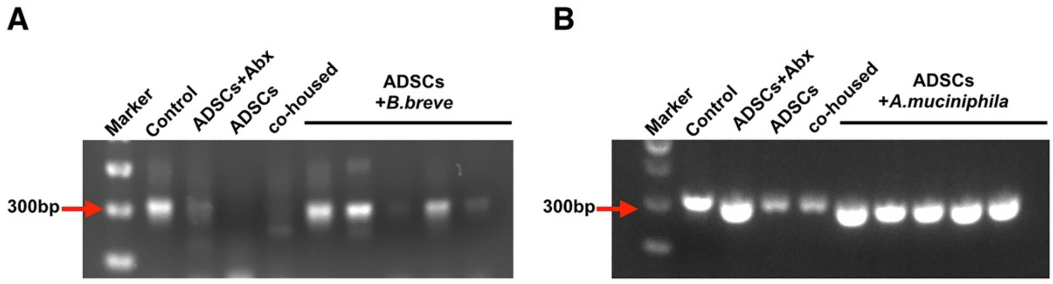

Figure S6. Colonization of *B.breve and A.muciniphila* after oral gavage. After one week oral gavage of *B.breve* and *A.muciniphila*, mice fecal DNA were extracted and PCR was performed using specific primer separately, amplified products were detected using agarose gel electrophoresis, (A) for *B.breve* (B) for *A.muciniphila*.
